# Supplementary material for: Role of UDP-Sugar Receptor P2Y14 in Murine Osteoblasts
Source: Int J Mol Sci. 2020 Apr 15;21(8):2747. doi: 10.3390/ijms21082747 (PMC7216066; doi:10.3390/ijms21082747)
Supplement: Supplementary file 1 [file ijms-21-02747-s001.pdf]

SUPPLEMENTAL MATERIALS
Role of UDP-sugar receptor $P2Y_{14}$ in murine osteoblasts

Nicholas Mikolajewicz*, Svetlana V. Komarova

Shriners Hospital for Children, Montreal, Canada
Faculty of Dentistry, McGill University, Montreal, Canada

Running title: $P2Y_{14}$ function in osteoblasts

Keywords: $P2Y_{14}$, GPR105, osteoblasts, bone, purinergic receptors

***Corresponding author**: Nicholas Mikolajewicz, Shriners Hospital for Children – Canada, Montreal, Quebec, Canada, H3G 1A6. Telephone: 647-878-1095; E-mail: Nicholas.mikolajewicz@mail.mcgill.ca

ORCID IDs:

Nicholas Mikolajewicz: https://orcid.org/0000-0002-7525-0384

Svetlana V. Komarova: https://orcid.org/0000-0003-3570-3147

Supplemental materials

Solutions and Reagents

Solutions. Phosphate-buffered saline (PBS; 140 mM NaCl, 3 mM KCl, 10 mM Na_2HPO_4, 2 mM KH_2PO_4, pH 7.4), autoclaved; Phosphate buffered saline with Tween 20 (PBST; PBS + 1% Tween 20); Physiological solution (PS; 130 mM NaCl, 5 mM KCl, 1 mM $MgCl_2$, 1 mM $CaCl_2$, 10 mM glucose, 20 mM HEPES, pH 7.6), sterilized by 0.2 µm filtration; RIPA lysis buffer (50 mM Tris, pH 7.4, 150 mM NaCl, 1% Nonidet P-40, 1 mM EDTA, 1 mg/mL aprotinin, 2 mg/mL leupeptin, 0.1 mM phenylmethylsulfonyl fluoride, 20 mM NaF, 0.5 mM Na3VO4); TBST buffer (10 mM Tris-HCL, pH 7.5, 150 mM NaCl, 1% Tween 20).

Reagents. Picro Sirius red stain kit (Cat. Ab150681) from Abcam. Anti-$P2Y_{14}$ receptor/GRP105 (extracellular) polyclonal rabbit antibody (Cat. APR-018) from Alomone Labs. High-capacity cDNA reverse transcription kit (Cat. 4368814); TaqMan Universal PCR Master Mix (Cat. 4304437); *Power* SYBR Green Master Mix (Cat. 4368702) from Applied Biosystems. Nitrocellulose membrane, 0.45 µm (Cat. 162-0115) from Bio-Rad. Cyclic AMP Select ELISA Kit (Cat. 501040) from Cayman Chemical. Phospho-p44/42 MAPK rabbit antibody (p-ERK1/2, Thr202/Tyr204; Cat. 9101); p44/42 MAPK rabbit antibody (ERK1/2; Cat. 9102); phospho-AMPKα rabbit antibody (Thr172; Cat. 2535); AMPKα rabbit antibody (Cat. 5831); phosphor-AMPKβ1 rabbit antibody (Ser182; Cat. 4186); AMPKβ1/2 rabbit antibody (Cat. 4150) from Cell Signalling Technology. Minimum essential medium α (αMEM; Cat. 12,000-022); Opti-MEM (Cat. 31985062) from Gibco. Alexa 647-conjuated phalloidin (Cat. A22287); 4',6-Diamidino-2-Phenylindole, Dihydrochloride (DAPI; Cat. D1306); D-Luciferin (Cat. L2916); Fura2-AM (Cat. F1221); Lipofectamine 3000 transfection reagent (Cat. L3000001); Quant-iT protein assay kit (Cat. Q33210) from Invitrogen. Puromycin (Cat. ant-pr-1) from InvivoGen. 35 mm glass-bottom dishes (Cat. P35G-1.5-14-C); 48-well glass-bottom plates (Cat. P48G-1.5-6-F) from MatTek Corporation. DNeasy Blood & Tissue Kit (Cat. 69506); QIAshredder columns (Cat. 79654); RNeasy Mini Kit (Cat. 74104) from Qiagen. Collagenase P from *Clostridium histolyticum* (Cat. 11213857001) from Roche. GPR105 ($P2Y_{14}$) Double Nickase Plasmid (Cat. sc-431244-NIC) from Santa Cruz. Adenosine 5'-triphosphate (ATP; Cat. A9187); Adenosine 5'-diphosphate (ADP; Cat. A2754); Fast red violet salt (Cat. F3381); L-ascorbic acid 2-phosphate sesquimagnesium salt hydrate (Cat. A8960); Naphthol AS-MX phosphate disodium salt (Cat. N5000); Uridine 5'-diphosphoglucose from *Saccharomyces cervisiae* (UDPG, microbial source; Cat. 94335); Venor GeM Mycoplasma PCR-based detection kit (Cat. MP0025) from Sigma-Aldrich. Pierce ECL western blotting substrate (Cat. 32106) from Thermo Scientific. PPTN hydrochloride (Cat. 4862) from Tocris. Dulbecco's modified eagle medium (DMEM; Cat. 319-020 CL); Fetal bovine serum (FBS; Cat. 080152); Penicillin streptomycin (Cat. 450-201-EL); Sodium pyruvate (Cat. 600-110-UL) from Wisent Bio Products. Collagenase Type II (Cat. LS004176) from Worthington Biochemical Corporation.

Supplemental Figures

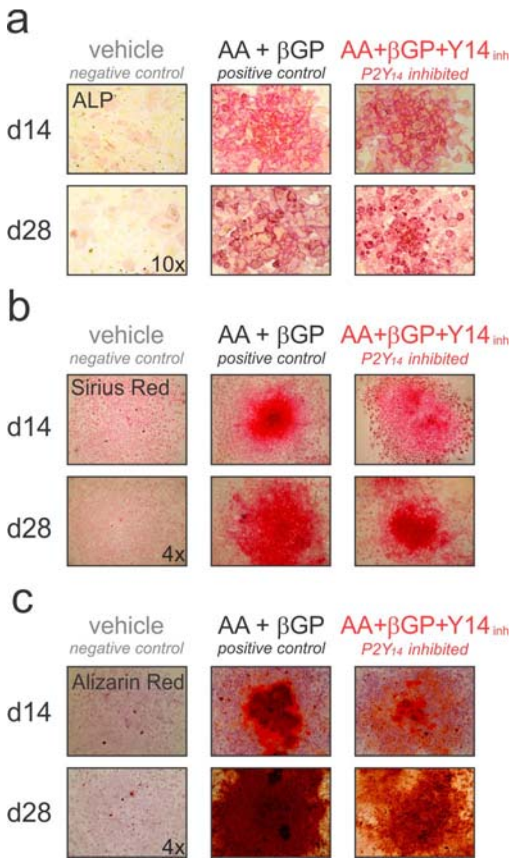

Supplemental Figure 1. Bone marrow-derived cells were cultured with or without osteogenic factors (50 µg/mL ascorbic acid, AA; 2 mM β-glycerol phosphate, βGP) and 100 nM PPTN ($Y14_{inh}$) and osteogenic phenotype was evaluated at days 14 and 28. **(a-c)** Cultures were fixed and stained for alkaline phosphatase (**a**; ALP), collagen (**b**; Sirius red stain) and mineralization (**c**; alizarin red stain). Stained cultures were visualized by brightfield microscopy.

Supplemental Tables

**Supplemental Table 1. Rapid review search strategies**	
**Review 1: P2Y$_{14}$ expression in bone**	
**Database**	Medline
**Search date**	Initial search: September 7th, 2018. Update search: April 2nd, 2020.
**Search Strategy**	("P2RY14" OR "GPR105" OR "P2Y14" OR "UDP-glucose receptor" OR "P2Y(14)" OR "P2RY(14)" OR "UDP-glucose" OR "UDP-g*" OR "PPTN" OR "MRS4174" OR "MRS4458" OR "MRS4478") AND ("osteoblast" OR "bone" OR "osteoclast" OR "osteocyte" OR "mesenchymal*" OR "MSC" OR "Bone and Bones"[MeSH Terms] OR "Osteoblasts"[MeSH Terms] OR "Osteoclasts"[MeSH Terms] OR "Mesenchymal Stromal Cells"[MeSH Terms])
**Inclusion criteria**	P2Y$_{14}$ expression (mRNA or protein) assessed in a bone-residing cell
**Hits**	43
**Eligible**	10 (6 from initial search, additional 3 from authors' library, additional 1 from update)<br>List of eligible studies provided in **Table 1**.
**Review 2: P2Y$_{14}$ response to endogenous ligands**	
**Database**	Medline
**Search date**	Initial search: July 13th 2018. Update search: April 2nd 2020.
**Search Strategy**	("P2RY14" OR "GPR105" OR "P2Y14" OR "P2Y(14)" OR "P2RY(14)") AND ("UDP-glucose receptor" OR "UDP-glucose" OR "UDP-g*" OR "UDP" OR "PPTN" OR "MRS4174" OR "MRS4458" OR "MRS4478")
**Inclusion criteria**	P2Y$_{14}$ response to endogenous ligand measured
**Hits**	87
**Eligible**	32<br>List of eligible studies provided in **Table S2**.

Supplemental Table 2. $P2Y_{14}$ EC_{50} review study-level characteristics.
FK, forskolin; HAS2, hyaluronan synthase 2; βHex, β-hexosaminidase; pERK1/2, ERK1/2 phosphorylation; UDP-Glu.A., UDP-glucuronic acid; UDP-Gal, UDP-galactose; UDPG, UDP-glucose.
*Studies included in meta-analysis ($[Ca^{2+}]_i$, cAMP, and IP_3 responses measured)
† Studies identified during literature search but excluded from meta-analysis due to lack of evidence of $P2Y_{14}$-dependent effects.

Study	Agonist	Species	Cell type	Response	$P2Y_{14}$ Source
Freeman 2001* [1]	UDPG, UDP-Gal, UDP-Glu.A.	Rat, mouse	HEK-293	$[Ca^{2+}]_i$ signaling	exogenous
Moore 2003* [2]	UDPG	Human	HEK-293	$[Ca^{2+}]_i$ signaling	exogenous
Skelton 2003* [3]	UDPG	Human	Immature monocyte-derived dendritic cells	$[Ca^{2+}]_i$ signaling	endogenous
Mamedova 2005† [4]	UDP	Human	U937 monocyte/ macrophages	$[Ca^{2+}]_i$ signaling	endogenous
Muller 2005 [5]	UDPG	Human	A649, BEAS-2B, AEC-II airway epithelial cells	IL8 release	endogenous
Scrivens 2005a* [6]	UDPG, UDP-Gal	Mouse	t-lymphocytes	cAMP, FK; proliferation	endogenous
Scrivens 2005b* [7]	UDPG	Human	U373MG astrocytoma	cAMP, FK	endogenous
Ault 2006 [8]	UDPG, UDP-Gal	Human	CY10569 yeast	B-galactosidase (binding assay)	exogenous
Light 2006† [9]	UDP	Mouse	Glial cells	$[Ca^{2+}]_i$ signaling	-
Scrivens 2006* [10]	UDPG	Human	Neutrophils	cAMP, FK; pERK1/2	endogenous
Ivanov 2007* [11]	UDPG, UDP-Gal, UDP-Glu.A.	Human	Cos-7	IP_3 accumulation	exogenous
Ko 2007* [12]	UDPG, UDP-Gal, UDP-Glu.A.	Human	Cos-7	IP_3 accumulation	exogenous
Brautigam 2008 [13]	UDPG	Mouse	N9 microglia	cAMP, Nitrate production	endogenous
Fricks 2008* [14]	UDP, UDPG	Human	Cos-7	IP_3 accumulation	exogenous
Shin 2008 [15]	UDPG	Human	plasmacytoid dendritic cells	IFNα production	endogenous
Arase 2009 [16]	UDPG	Human	Ishikawa 3-H-12 adenocarcinoma	IL8 release	endogenous
Carter 2009* [17]	UDP, UDPG	Human	HEK-293, CHO, C6 glioma	cAMP, FK	exogenous
Fricks 2009* [18]	UDPG, UDP-gal, UDP-glu.A	Human	HEK-293, C6 glioma	cAMP, FK; pERK1/2	exogenous
Das 2010* [19]	UDP, UDPG	Human	HEK-293	cAMP, FK	exogenous
Gao 2010* [20]	UDP, UDPG	Rat	RBL-2H3 mast cells	$[Ca^{2+}]_i$ signaling; GTP-binding; βHex release	endogenous
Hamel 2011* [21]	UDP, UDPG, UDP-Gal, UDP-Glu.A.	Human	HEK-293	$[Ca^{2+}]_i$ signaling	exogenous
Sesma 2012 [22]	UDPG	Human	undifferentiated and differentiated HL60 neutrophils	Chemotaxis; RhoA activation	endogenous

Barret 2013* [23]	UDPG	Human	C6 glioma	cAMP, FK	exogenous
Alsaqati 2014 [24]	UDPG	Porcine	Pancreatic artery	contraction	endogenous
Haanes 2014 [25]	UDPG	Mouse	Coronary artery, basilar artery	contraction	endogenous
Jokela 2014 [26]	UDPG	Human	HaCaT keratinocytes	HAS2 expression	endogenous
Azroyan 2015 [27]	UDPG	Canine	MDCK-C11 kidney cells	Ligand-binding assay	endogenous
Kiselev 2015* [28]	UDP, UDPG, UDP-Glu.A.	Human	CHO	cAMP, FK; flow cytometry tracer (binding assay)	exogenous
Trujillo 2015* [29]	UDP, UDPG	Human	Cos-7	IP_3 accumulation	exogenous
Gendaszewska-Darmach 2016* [30]	UDPG	Rat	RBL-2H3 mast cells	$[Ca^{2+}]_i$ signaling; βHex release	endogenous
Abbas 2018 [31]	UDPG, UDP-Glu.A.	Porcine	Coronary artery	contraction	endogenous
Lin 2019 [32]	UDPG	Rat	Satellite glial cells	IL-1β secretion<br>CCL2 secretion<br>Viability<br>pERK, pp-38, pJNK	endogenous

Supplemental Table 3. **Meta-regression analysis of $P2Y_{14}$ EC_{50}.** Rapid review was conducted to identify studies evaluating the dose-response of $P2Y_{14}$ to various uridine-agonists, and data was extracted and fit to hill functions to estimate the EC_{50}. Meta-regression analysis summarizes the relationship between uridine agonist and the observed EC_{50}, controlling for $P2Y_{14}$ source, $P2Y_{14}$ species and the type of measured response.
FK, forskolin; SE, standard error; Z, z-score; 95% CI, 95% confidence intervals.

	**Coefficient ($log_{10}M$)**	**SE**	**Z**	**p**	**95% CI**
Intercept	-7.08	0.12	-59.29	<0.001	-7.32, -6.85
**$P2Y_{14}$ source**					
exogenous	(ref)	-	-	-	-
endogenous	2.45	0.23	10.85	<0.001	2.01, 2.90
**$P2Y_{14}$ Species**					
Human	(ref)	-	-	-	-
Mouse	0.53	0.31	1.72	0.09	-0.07, 1.13
Rat	0.72	0.25	2.85	0.004	0.23, 1.22
**Measured Response**					
cAMP (FK-induced)	(ref)	-	-	-	-
$[Ca^{2+}]_i$ elevation	-0.95	0.18	-5.42	<0.001	-1.30, -0.61
IP_3 Accumulation	0.68	0.16	4.21	<0.001	0.37, 1.00
**Agonist**					
UDP Glucose	(ref)	-	-	-	-
UDP	-0.32	0.18	-1.76	0.08	-0.68, 0.04
UDP Galactose	-0.01	0.19	-0.07	0.94	-0.38, 0.36
UDP Glucoronic Acid	-0.02	0.18	-0.09	0.93	-0.37, 0.33
**Heterogeneity Statistics**					
Q_{model} (heterogeneity explained by model)				180 (df = 8), p<0.001	
$Q_{residual}$ (residual heterogeneity)				1030 (df = 45), p<0.001	
τ^2 (residual heterogeneity)				0.18	
I^2 (residual heterogeneity/unaccounted variance)				95.5%	
$R^2_{explained}$ (amount of heterogeneity explained by model)				80.1%	

Supplemental Table 4. Primer, Probe, and PAM sequences		
**Target**	**Description**	**Sequence (5' – 3') / Probe ID**
P2ry14	CRISPR/Cas9; PAM sequence	Strand A: CATTCCCGTGTTGTACGGTA<br>Strand B: CTTTGTGATCAGGGTGTTCC
P2ry14	Touchdown PCR; flanking primers	Fwd: AGGCCCATGAGAAAGTCAGC<br>Rv: ACAACTCCACCACCACAGAC
P2ry14	qRT-PCR; primers	Fwd: CCACCACAGACCCTCCAAAC<br>Rv: CAACACGGGAATGATCTGCTTT
Run2x	qRT-PCR; primers	Fwd: TGGCTTGGGTTTCAGGTTAG<br>Rv: TCGGTTTCTTAGG-GTCTTGGA
Opn	qRT-PCR; primers	Fwd: GTGGACTC-GGATGAATCTG<br>Rv: TCGACTGTAGGGACGATTG
Gapdh	qRT-PCR; primers	Fwd: CAAGTATGATGACATCAAGAAGGTGG<br>Rv: GGAAGA-GTGGGAGTTGCTGTTG
Dmp1	qRT-PCR; probe	Mm01208363_m1
Sost	qRT-PCR; probe	Mm00470479_m1
Cola1	qRT-PCR; probe	Mm00801666_g1
Osx	qRT-PCR; probe	Mm00504574_m1
Gapdh	qRT-PCR; probe	Mm99999915_g1

Supplemental Table 5. PCR cycling conditions		
**Stage**	**Description**	**Cycles (n)**
**Touchdown PCR (gDNA template)**		
**Phase 1**	Denaturation: 94 °C, 180 s	1
**Phase 2**	Denaturation: 94 °C, 30 s	10
	Annealing: 65 to 55 °C (incremented by -1 °C / cycle), 45 s	
	Elongation: 72 °C, 60 s	
**Phase 3**	Denaturation 94 °C, 30 s	25
	Annealing: 55 °C, 45 s	
	Elongation: 72 C, 60 s	
**Phase 4**	Elongation: 72 °C, 600 s	1
	Hold: 4 °C	
**qRT-PCR (cDNA template)**		
**Phase 1**	Denaturation: 94 °C, 180 s	1
**Phase 2**	Denaturation 94 °C, 30 s	40
	Annealing: 60 °C, 45 s	
	Elongation: 72 C, 60 s	
**Phase 3**	Elongation: 72 °C, 600 s	1
	Hold: 4 °C	

References

1. Freeman, K.; Tsui, P.; Moore, D.; Emson, P.C.; Vawter, L.; Naheed, S.; Lane, P.; Bawagan, H.; Herrity, N.; Murphy, K., et al. Cloning, pharmacology, and tissue distribution of G-protein-coupled receptor GPR105 (KIAA0001) rodent orthologs. *Genomics* **2001**, *78*, 124-128, doi:10.1006/geno.2001.6662.
2. Moore, D.J.; Murdock, P.R.; Watson, J.M.; Faull, R.L.; Waldvogel, H.J.; Szekeres, P.G.; Wilson, S.; Freeman, K.B.; Emson, P.C. GPR105, a novel Gi/o-coupled UDP-glucose receptor expressed on brain glia and peripheral immune cells, is regulated by immunologic challenge: possible role in neuroimmune function. *Brain research. Molecular brain research* **2003**, *118*, 10-23.
3. Skelton, L.; Cooper, M.; Murphy, M.; Platt, A. Human immature monocyte-derived dendritic cells express the G protein-coupled receptor GPR105 (KIAA0001, P2Y14) and increase intracellular calcium in response to its agonist, uridine diphosphoglucose. *Journal of immunology (Baltimore, Md. : 1950)* **2003**, *171*, 1941-1949.
4. Mamedova, L.; Capra, V.; Accomazzo, M.R.; Gao, Z.G.; Ferrario, S.; Fumagalli, M.; Abbracchio, M.P.; Rovati, G.E.; Jacobson, K.A. CysLT1 leukotriene receptor antagonists inhibit the effects of nucleotides acting at P2Y receptors. *Biochemical pharmacology* **2005**, *71*, 115-125, doi:10.1016/j.bcp.2005.10.003.
5. Muller, T.; Bayer, H.; Myrtek, D.; Ferrari, D.; Sorichter, S.; Ziegenhagen, M.W.; Zissel, G.; Virchow, J.C., Jr.; Luttmann, W.; Norgauer, J., et al. The P2Y14 receptor of airway epithelial cells: coupling to intracellular Ca2+ and IL-8 secretion. *American journal of respiratory cell and molecular biology* **2005**, *33*, 601-609, doi:10.1165/rcmb.2005-0181OC.
6. Scrivens, M.; Dickenson, J.M. Functional expression of the P2Y14 receptor in murine T-lymphocytes. *British journal of pharmacology* **2005**, *146*, 435-444, doi:10.1038/sj.bjp.0706322.
7. Scrivens, M.; Dickenson, J.M. Pharmacological effects mediated by UDP-glucose that are independent of P2Y14 receptor expression. *Pharmacological research* **2005**, *51*, 533-538, doi:10.1016/j.phrs.2005.02.001.
8. Ault, A.D.; Broach, J.R. Creation of GPCR-based chemical sensors by directed evolution in yeast. *Protein engineering, design & selection : PEDS* **2006**, *19*, 1-8, doi:10.1093/protein/gzi069.
9. Light, A.R.; Wu, Y.; Hughen, R.W.; Guthrie, P.B. Purinergic receptors activating rapid intracellular Ca increases in microglia. *Neuron glia biology* **2006**, *2*, 125-138, doi:10.1017/s1740925x05000323.
10. Scrivens, M.; Dickenson, J.M. Functional expression of the P2Y14 receptor in human neutrophils. *European journal of pharmacology* **2006**, *543*, 166-173, doi:10.1016/j.ejphar.2006.05.037.
11. Ivanov, A.A.; Fricks, I.; Kendall Harden, T.; Jacobson, K.A. Molecular dynamics simulation of the P2Y14 receptor. Ligand docking and identification of a putative binding site of the distal hexose moiety. *Bioorganic & medicinal chemistry letters* **2007**, *17*, 761-766, doi:10.1016/j.bmcl.2006.10.081.
12. Ko, H.; Fricks, I.; Ivanov, A.A.; Harden, T.K.; Jacobson, K.A. Structure-activity relationship of uridine 5'-diphosphoglucose analogues as agonists of the human P2Y14 receptor. *Journal of medicinal chemistry* **2007**, *50*, 2030-2039, doi:10.1021/jm061222w.

13. Brautigam, V.M.; Dubyak, G.R.; Crain, J.M.; Watters, J.J. The inflammatory effects of UDP-glucose in N9 microglia are not mediated by P2Y14 receptor activation. *Purinergic signalling* **2008**, *4*, 73-78, doi:10.1007/s11302-008-9095-1.
14. Fricks, I.P.; Maddileti, S.; Carter, R.L.; Lazarowski, E.R.; Nicholas, R.A.; Jacobson, K.A.; Harden, T.K. UDP is a competitive antagonist at the human P2Y14 receptor. *The Journal of pharmacology and experimental therapeutics* **2008**, *325*, 588-594, doi:10.1124/jpet.108.136309.
15. Shin, A.; Toy, T.; Rothenfusser, S.; Robson, N.; Vorac, J.; Dauer, M.; Stuplich, M.; Endres, S.; Cebon, J.; Maraskovsky, E., et al. P2Y receptor signaling regulates phenotype and IFN-alpha secretion of human plasmacytoid dendritic cells. *Blood* **2008**, *111*, 3062-3069, doi:10.1182/blood-2007-02-071910.
16. Arase, T.; Uchida, H.; Kajitani, T.; Ono, M.; Tamaki, K.; Oda, H.; Nishikawa, S.; Kagami, M.; Nagashima, T.; Masuda, H., et al. The UDP-glucose receptor P2RY14 triggers innate mucosal immunity in the female reproductive tract by inducing IL-8. *Journal of immunology (Baltimore, Md. : 1950)* **2009**, *182*, 7074-7084, doi:10.4049/jimmunol.0900001.
17. Carter, R.L.; Fricks, I.P.; Barrett, M.O.; Burianek, L.E.; Zhou, Y.; Ko, H.; Das, A.; Jacobson, K.A.; Lazarowski, E.R.; Harden, T.K. Quantification of Gi-mediated inhibition of adenylyl cyclase activity reveals that UDP is a potent agonist of the human P2Y14 receptor. *Molecular pharmacology* **2009**, *76*, 1341-1348, doi:10.1124/mol.109.058578.
18. Fricks, I.P.; Carter, R.L.; Lazarowski, E.R.; Harden, T.K. Gi-dependent cell signaling responses of the human P2Y14 receptor in model cell systems. *The Journal of pharmacology and experimental therapeutics* **2009**, *330*, 162-168, doi:10.1124/jpet.109.150730.
19. Das, A.; Ko, H.; Burianek, L.E.; Barrett, M.O.; Harden, T.K.; Jacobson, K.A. Human P2Y(14) receptor agonists: truncation of the hexose moiety of uridine-5'-diphosphoglucose and its replacement with alkyl and aryl groups. *Journal of medicinal chemistry* **2010**, *53*, 471-480, doi:10.1021/jm901432g.
20. Gao, Z.G.; Ding, Y.; Jacobson, K.A. UDP-glucose acting at P2Y14 receptors is a mediator of mast cell degranulation. *Biochemical pharmacology* **2010**, *79*, 873-879, doi:10.1016/j.bcp.2009.10.024.
21. Hamel, M.; Henault, M.; Hyjazie, H.; Morin, N.; Bayly, C.; Skorey, K.; Therien, A.G.; Mancini, J.; Brideau, C.; Kargman, S. Discovery of novel P2Y14 agonist and antagonist using conventional and nonconventional methods. *Journal of biomolecular screening* **2011**, *16*, 1098-1105, doi:10.1177/1087057111415525.
22. Sesma, J.I.; Kreda, S.M.; Steinckwich-Besancon, N.; Dang, H.; Garcia-Mata, R.; Harden, T.K.; Lazarowski, E.R. The UDP-sugar-sensing P2Y(14) receptor promotes Rho-mediated signaling and chemotaxis in human neutrophils. *American journal of physiology. Cell physiology* **2012**, *303*, C490-498, doi:10.1152/ajpcell.00138.2012.
23. Barrett, M.O.; Sesma, J.I.; Ball, C.B.; Jayasekara, P.S.; Jacobson, K.A.; Lazarowski, E.R.; Harden, T.K. A selective high-affinity antagonist of the P2Y14 receptor inhibits UDP-glucose-stimulated chemotaxis of human neutrophils. *Molecular pharmacology* **2013**, *84*, 41-49, doi:10.1124/mol.113.085654.
24. Alsaqati, M.; Latif, M.L.; Chan, S.L.; Ralevic, V. Novel vasocontractile role of the P2Y(1)(4) receptor: characterization of its signalling in porcine isolated pancreatic arteries. *British journal of pharmacology* **2014**, *171*, 701-713, doi:10.1111/bph.12473.

25. Haanes, K.A.; Edvinsson, L. Characterization of the contractile P2Y14 receptor in mouse coronary and cerebral arteries. *FEBS letters* **2014**, *588*, 2936-2943, doi:10.1016/j.febslet.2014.05.044.
26. Jokela, T.A.; Karna, R.; Makkonen, K.M.; Laitinen, J.T.; Tammi, R.H.; Tammi, M.I. Extracellular UDP-glucose activates P2Y14 Receptor and Induces Signal Transducer and Activator of Transcription 3 (STAT3) Tyr705 phosphorylation and binding to hyaluronan synthase 2 (HAS2) promoter, stimulating hyaluronan synthesis of keratinocytes. *The Journal of biological chemistry* **2014**, *289*, 18569-18581, doi:10.1074/jbc.M114.551804.
27. Azroyan, A.; Cortez-Retamozo, V.; Bouley, R.; Liberman, R.; Ruan, Y.C.; Kiselev, E.; Jacobson, K.A.; Pittet, M.J.; Brown, D.; Breton, S. Renal intercalated cells sense and mediate inflammation via the P2Y14 receptor. *PloS one* **2015**, *10*, e0121419, doi:10.1371/journal.pone.0121419.
28. Kiselev, E.; Balasubramanian, R.; Uliassi, E.; Brown, K.A.; Trujillo, K.; Katritch, V.; Hammes, E.; Stevens, R.C.; Harden, T.K.; Jacobson, K.A. Design, synthesis, pharmacological characterization of a fluorescent agonist of the P2Y(1)(4) receptor. *Bioorganic & medicinal chemistry letters* **2015**, *25*, 4733-4739, doi:10.1016/j.bmcl.2015.08.021.
29. Trujillo, K.; Paoletta, S.; Kiselev, E.; Jacobson, K.A. Molecular modeling of the human P2Y14 receptor: A template for structure-based design of selective agonist ligands. *Bioorganic & medicinal chemistry* **2015**, *23*, 4056-4064, doi:10.1016/j.bmc.2015.03.042.
30. Gendaszewska-Darmach, E.; Weglowska, E.; Walczak-Drzewiecka, A.; Karas, K. Nucleoside 5'-O-monophosphorothioates as modulators of the P2Y14 receptor and mast cell degranulation. *Oncotarget* **2016**, *7*, 69358-69370, doi:10.18632/oncotarget.12541.
31. Abbas, Z.S.B.; Latif, M.L.; Dovlatova, N.; Fox, S.C.; Heptinstall, S.; Dunn, W.R.; Ralevic, V. UDP-sugars activate P2Y14 receptors to mediate vasoconstriction of the porcine coronary artery. *Vascular pharmacology* **2018**, *103-105*, 36-46, doi:10.1016/j.vph.2017.12.063.
32. Lin, J.; Liu, F.; Zhang, Y.Y.; Song, N.; Liu, M.K.; Fang, X.Y.; Liao, D.Q.; Zhou, C.; Wang, H.; Shen, J.F. P2Y14 receptor is functionally expressed in satellite glial cells and mediates interleukin-1beta and chemokine CCL2 secretion. *J Cell Physiol* **2019**, *234*, 21199-21210, doi:10.1002/jcp.28726.
